# Monoglyceride Lipase Deficiency Is Associated with Altered Thrombogenesis in Mice

**DOI:** 10.3390/ijms24043116

**Published:** 2023-02-04

**Authors:** Madeleine Goeritzer, Katharina B. Kuentzel, Sarah Beck, Melanie Korbelius, Silvia Rainer, Ivan Bradić, Dagmar Kolb, Marion Mussbacher, Waltraud C. Schrottmaier, Alice Assinger, Axel Schlagenhauf, René Rost, Benjamin Gottschalk, Thomas O. Eichmann, Thomas Züllig, Wolfgang F. Graier, Nemanja Vujić, Dagmar Kratky

**Affiliations:** 1 Gottfried Schatz Research Center, Molecular Biology and Biochemistry, Medical University of Graz, 8010 Graz, Austria; 2 Institute of Vascular Biology and Thrombosis Research, Center for Physiology and Pharmacology, Medical University of Vienna, 1190 Vienna, Austria; 3 Institute of Experimental Biomedicine, University Hospital Würzburg and Rudolf Virchow Center for Integrative and Translational Bioimaging, University of Würzburg, 97080 Würzburg, Germany; 4 Core Facility Ultrastructural Analysis, Medical University of Graz, 8010 Graz, Austria; 5 BioTechMed-Graz, 8010 Graz, Austria; 6 Department of Pharmacology and Toxicology, University of Graz, 8010 Graz, Austria; 7 Department of General Pediatrics and Adolescent Medicine, Medical University of Graz, 8010 Graz, Austria; 8 Institute of Molecular Biosciences, University of Graz, 8010 Graz, Austria; 9 Core Facility Mass Spectrometry, Medical University of Graz, 8010 Graz, Austria

**Keywords:** platelets, MGL, in vitro and in vivo thrombus formation, platelet activation, platelet aggregation

## Abstract

Monoglyceride lipase (MGL) hydrolyzes monoacylglycerols (MG) to glycerol and one fatty acid. Among the various MG species, MGL also degrades 2-arachidonoylglycerol, the most abundant endocannabinoid and potent activator of the cannabinoid receptors 1 and 2. We investigated the consequences of MGL deficiency on platelet function using systemic (Mgl^−/−^) and platelet-specific Mgl-deficient (platMgl^−/−^) mice. Despite comparable platelet morphology, loss of MGL was associated with decreased platelet aggregation and reduced response to collagen activation. This was reflected by reduced thrombus formation in vitro, accompanied by a longer bleeding time and a higher blood volume loss. Occlusion time after FeCl_3_-induced injury was markedly reduced in Mgl^−/−^ mice, which is consistent with contraction of large aggregates and fewer small aggregates in vitro. The absence of any functional changes in platelets from platMgl^−/−^ mice is in accordance with lipid degradation products or other molecules in the circulation, rather than platelet-specific effects, being responsible for the observed alterations in Mgl^−/−^ mice. We conclude that genetic deletion of MGL is associated with altered thrombogenesis.

## 1. Introduction

Prevention of bleeding is the primary function of platelets, but they can also recruit leukocytes and progenitor cells to sites of vascular injury and inflammation, and release pro- and anti-inflammatory as well as pro-angiogenic factors and microparticles into the circulation. Due to their diverse functions, platelets are also involved in the etiology of various pathologies, such as diabetes, cardiovascular and autoimmune diseases [1,2,3,4]. In particular, previous findings from studies of human platelets in vitro and in animal models in vivo demonstrated that platelets play a central role in atherosclerosis and atherothrombosis [5].

Monoglyceride lipase (MGL) hydrolyzes monoacylglycerols (MG) to complete the final step of intracellular lipolysis—the degradation of triacylglycerol (TG) to yield glycerol and fatty acids (FA) [6]. The degradation of phospholipids by diacylglycerol lipase also leads to the formation of MG [7] as substrates for MGL-mediated hydrolysis. The resulting FA have a variety of functions, being the most efficient energy substrates, precursors for lipid and membrane synthesis, and ligands for signaling processes. One of the MG species hydrolyzed by MGL is 2-arachidonoylglycerol (2-AG), the most abundant endogenous ligand for cannabinoid receptors (CBR) 1 and 2 [8,9]. Endocannabinoids are essential regulators of hematopoiesis, platelet aggregation, apoptosis, chemokine release [10,11], and several pathophysiological responses [12,13]. Among blood cells, platelets represent an important source of circulating endocannabinoids, particularly 2-AG, which may be involved in several pathophysiological responses [12,13]. 2-AG stimulates platelets through an MGL-triggered mechanism leading to free AA and its metabolization by platelet cyclooxygenase-1/thromboxane synthase to TXA2 independently of CB receptors. This indicates that MGL inhibition may protect platelets from activation by high levels of endocannabinoids and that pharmacological CBR1 and CBR2 ligands will have no effect on platelets and platelet-dependent progression and complications of cardiovascular disease [14].Platelets can be targeted by endocannabinoid signaling as they express both CBR1 and CBR2 [15]. 2-AG is a true agonist of human platelets [16], as it stimulates platelet formation and release, thereby counteracting thrombocytopenia-associated diseases [17]. Moreover, 2-AG activates platelets, triggering platelet shape change, aggregation, and secretion in a dose-dependent manner, which can be reversed by CBR1 and CBR2 antagonists [18,19]. Due to incomplete lipolysis, mice globally lacking MGL accumulate MG (including the endocannabinoid 2-AG) in multiple cells and tissues [20,21], resulting in reduced FA availability. In contrast, MGL-triggered 2-AG degradation and release of arachidonic acid (AA) also stimulates platelets. Therefore, MGL inhibition may protect platelets from activation by reducing AA availability [14], thus counteracting the pro-aggregatory action of 2-AG. Of note, loss of MGL positively affects atherosclerotic plaque stability and reduces macrophage foam cell formation in mice [21].

According to genome-wide RNA-sequencing data from humans [22] and our previously published results in mice [23], MGL is expressed in platelets. Here, we hypothesized that MGL is critical for the generation of lipid mediators that play an essential role in hemostasis. To investigate the role of MGL in platelets, we studied the effect of genomic and platelet-specific (plat) MGL knock-out (Mgl^−/−^) on platelet morphology and aggregation. Our experiments revealed that most MG species, including AG, are highly increased in platelets of Mgl^−/−^ mice. Mgl^−/−^ mice displayed reduced thrombus formation in vitro, accompanied by a prolonged tail bleeding time and a higher blood volume loss. Vessel occlusion time after FeCl_3_-induced injury was markedly reduced in Mgl^−/−^ mice, consistent with larger platelet-rich aggregates in vitro. In addition, platelet aggregation and activation were affected only in systemic Mgl^−/−^, but not in platMgl^−/−^ mice, indicating that the availability of plasma-derived factors likely modulates thrombus formation and hemostasis.

## 2. Results

### 2.1. Unaltered Morphology and Spreading in Mgl^−/−^ Platelets

**Figure 1:**
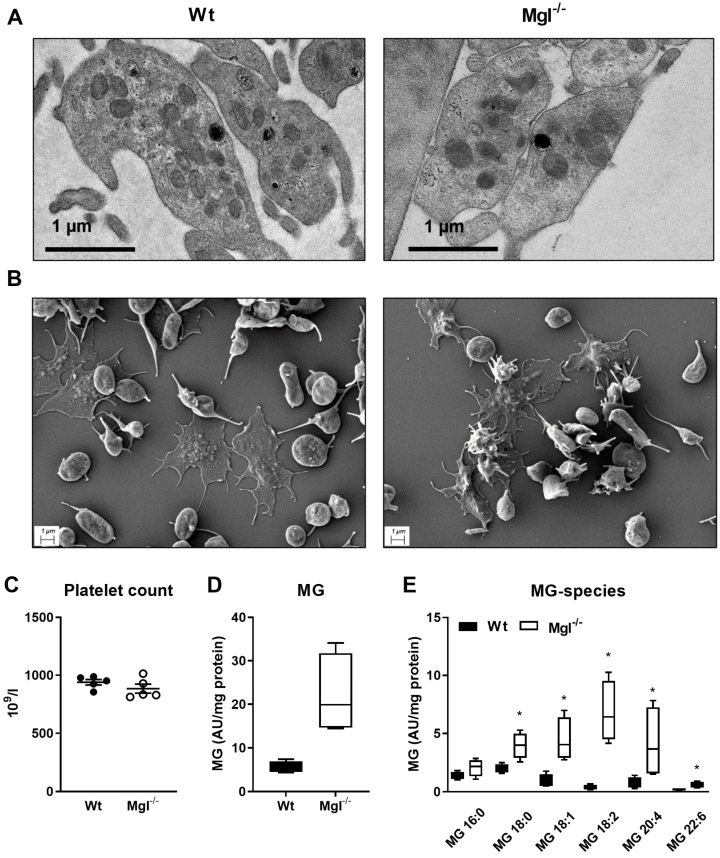
Unchanged morphology and spreading, but increased MG species in Mgl^−/−^ platelets. (**A**) Representative electron micrographs of platelets from Wt and Mgl^−/−^ mice. (**B**) Platelet spreading was visualized by scanning electron microscopy. (**C**) Platelet counts in blood from Wt and Mgl^−/−^ mice were measured using an automated cell counter. Data represent single values and mean ± SEM (n = 5). Concentrations of (**D**) monoglycerides (MG) and (**E**) various MG species in purified platelets isolated from Wt and Mgl^−/−^ mice were determined by UPLC-MS. Data are expressed as median ± IQR (n = 4, platelets from 3–4 mice were pooled per sample). *, *p* < 0.05. Significance was calculated by Mann–Whitney U test.

Platelet spreading, the process by which adherent platelets first flatten at sites of vascular injury and increase their contact area by deformation of the plasma membrane, has been recognized as a crucial step for hemostasis and thrombosis [24]. Despite profound MGL expression in mouse platelets [23], loss of MGL did not reveal any significant structural or morphological alterations in platelets as visualized by transmission and scanning electron microscopy of these from Mgl^−/−^ and Wt mice (Figure 1A,B). The platelet counts were comparable between the genotypes (Figure 1C).

The lipid composition of membranes defines platelet activities, such as membrane fluidity, eicosanoid generation, and signaling. In platelets from Mgl^−/−^ mice, we measured an increased concentration of MG by targeted lipidomics (Figure 1D). Lipid species analysis revealed that several saturated and unsaturated MG species, namely, MG 18:0, 18:1, 18:2, 20:4, and 22:6, were significantly increased in Mgl^−/−^ platelets (Figure 1E). One of the most abundant MG species in Mgl^−/−^ platelets was MG 20:4, representing the AG species. We also found alterations in levels of distinct phosphatidylcholine (PC), phosphatidylethanolamine (PE), lysophosphatidylcholine (LPC), and lysophosphatidylethanolamine (LPE) species, and increased ceramides (Cer), whereas levels of neutral lipids (such as TG, diacylglycerol (DG), and cholesteryl esters (CE)) remained unchanged (Figure S1A–K).

### 2.2. Increased Mitochondrial Respiration in Mgl^−/−^ Platelets

**Figure 2:**
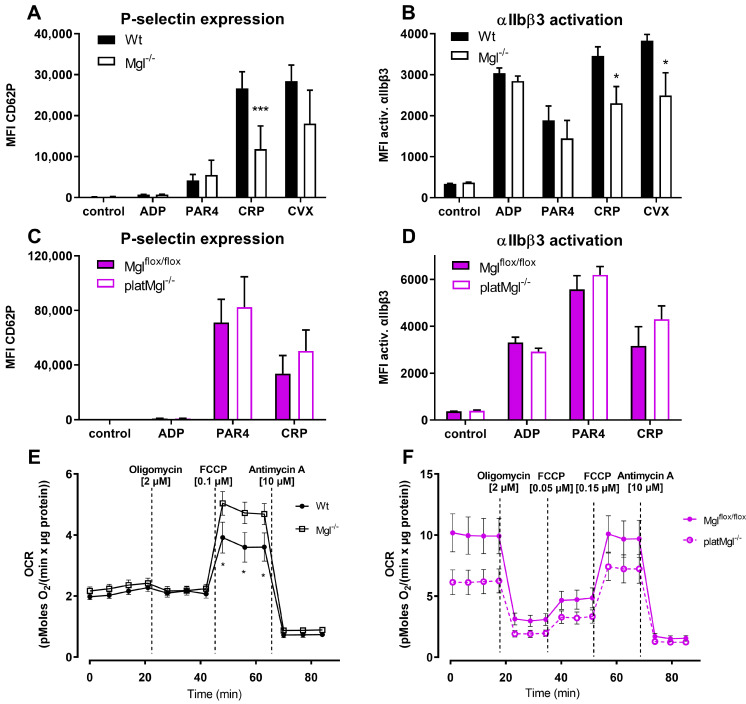
Increased maximal mitochondrial respiration but decreased activation in platelet from Mgl^−/−^ mice. Blood from (**A**,**B**) Mgl^−/−^ and (**C**,**D**) platMgl^−/−^ mice was activated with ADP (50 µM), protease-activated receptor 4 peptide (PAR4, 75 µM), collagen-related peptide (CRP, 10 µg/mL), and convulxin (CVX, 125 ng/mL) in the presence of (**A**,**C**) a PE-Cy7-conjugated anti-mouse P-selectin antibody or (**B**,**D**) a JON/A-PE antibody directed against the activated form of mouse integrin αIIbβ3. Data are expressed as geometric mean values of fluorescence intensity (MFI) + SEM (n = 8). *, *p* < 0.05, ***, *p* ≤ 0.001. Oxygen consumption rate (OCR) in isolated platelets from (**E**) Mgl^−/−^ and (**F**) platMgl^−/−^ mice and the respective controls were determined using a Seahorse XF Analyzer. Ten million cells were seeded in XF assay medium supplemented with sodium pyruvate (1 mM), L-glutamine (2 mM), and glucose (25 mM) per 96-well. Cells were treated with oligomycin, carbonyl cyanide-4-(trifluoromethoxy)phenylhydrazone (FCCP), and antimycin A. Values were normalized to protein content using the Pierce^TM^ BCA protein assay kit according to the manufacturer’s instructions. Data are presented as mean values ± SEM of sextuplicates from 5 independent experiments. *, *p* < 0.05. Significance was calculated by ANOVA followed by Bonferroni post hoc test.

To further investigate the reduced hemostatic function of platelets in Mgl^−/−^ mice, we incubated blood from Wt and Mgl^−/−^ mice with various compounds that induce platelet activation and performed flow cytometric analyses of P-selectin expression and integrin αIIbβ3 activation. Blood from Mgl^−/−^ mice showed reduced P-selectin expression and integrin αIIbβ3 activation in response to collagen-related peptide (CRP), which mimics the structure of collagen and acts as a strong platelet agonist via GPVI. Integrin αIIbβ3 activation was also significantly reduced in response to convulxin (CVX), which potently activates platelets via the ITAM-coupled receptor GPVI [25]. Responses to ADP and protease-activated receptor 4 (PAR-4) peptide, both stimulating platelets via GPCR-coupled receptors, revealed no differences between integrin activation and P-selectin exposure in Mgl^−/−^ vs. control platelets (Figure 2A, B). To clarify whether systemic or platelet-specific effects were responsible for reduced platelet activation, we generated platMgl^−/−^ mice. These mice also had a comparable number of platelets (Figure S2). Regardless of agonist treatments, however, comparable results between platMgl^−/−^ and control mice (Figure 2C,D) argue against a platelet-intrinsic effect on their activation.

Since mitochondrial respiration is of particular importance for platelet activation [26] and mitochondrial damage or dysfunction markedly impairs platelet function and survival [26], we performed a mitochondrial stress test to examine oxygen consumption rate (OCR) in platelets. In contrast to our expectations, we detected increased maximal OCR in platelets of Mgl^−/−^ mice (Figure 2E), whereas OCR of platelets from platMgl^−/−^ mice was not significantly different from controls (Figure 2F).

These results suggest that the reduced platelet activation is mediated selectively through the GPVI receptor pathway, at least at the given reagent concentrations, and that platelet activation remains unaltered upon platelet-specific MGL deficiency. Impaired platelet activation in Mgl^−/−^ mice might be independent of mitochondrial function because platelets from Mgl^−/−^ mice have increased rather than decreased maximal respiration.

### 2.3. Loss of MGL Affects Thrombus Formation In Vitro

**Figure 3:**
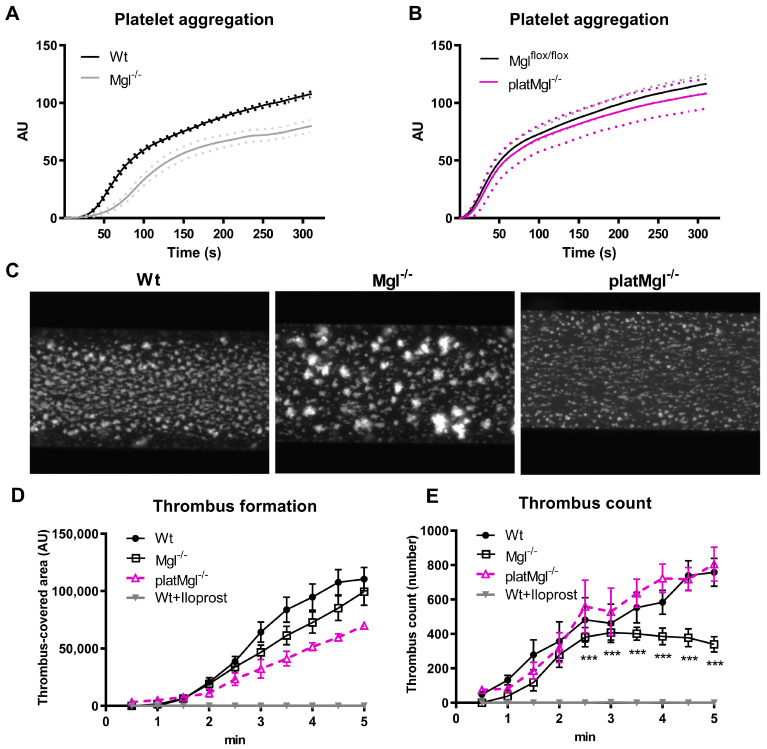
Reduced in vitro thrombus formation in blood from Mgl^−/−^, but not platMgl^−/−^, mice. Platelet aggregation in (**A**) Mgl^−/−^ and (**B**) platMgl^−/−^ blood was measured using a Multiplate^®^ analyzer. Data are expressed as mean arbitrary units (AU) (n = 7, 6). (**C**) Platelet reactivity was determined by an in vitro thrombus formation assay in which platelets were stained in whole blood and perfused over collagen-coated channels. Representative images of in vitro thrombus formation of Wt, Mgl^−/−^, and platMgl^−/−^ blood recorded by fluorescence microscopy; magnification, ×10. Computerized image analysis was used to determine (**D**) the area covered with thrombi and (**E**) the number of thrombi between 1 and 5 min of perfusion. Iloprost, which inhibits thrombus formation, was used as a negative control in Wt blood. Data are expressed in arbitrary units (AU) (mean ± SEM) (n = 5–6). ***, *p* ≤ 0.001.

The ability of platelets to form a hemostatic plug depends on their aggregation. To assess platelet function, we performed whole blood impedance aggregometry, which revealed that collagen-induced platelet aggregation was reduced in platelets from Mgl^−/−^ (Figure 3A) but not from platMgl^−/−^ mice (Figure 3B).

To study platelet reactivity under flow conditions, which mimics the in vivo situation, we perfused whole blood of Mgl^−/−^ and platMgl^−/−^ mice over collagen-coated channels and recorded thrombus formation by fluorescence microscopy as previously described [23]. Fluorescence images showed clear differences in aggregate formation between Wt and Mgl^−/−^ mice, with platelets from Mgl^−/−^ mice appearing much brighter and more contracted (Figure 3C). Thrombus-covered area and thrombi counts were calculated by computerized image analysis using Cellix VenaFlux software and a costumed ImageJ macro. We observed no significant difference in surface coverage in Mgl^−/−^ and platMgl^−/−^ blood compared to control mice between 2.5 and 5 min of perfusion (Figure 3D). Iloprost, a structural analog of prostacyclin, was used as a control to inhibit thrombus formation. Due to the larger aggregates involving more platelets, fewer small aggregates formed in the blood of Mgl^−/−^ mice (Figure 3E).

### 2.4. Reduced Hemostatic Function in Mgl^−/−^ but Not in platMgl^−/−^ Mice

**Figure 4:**
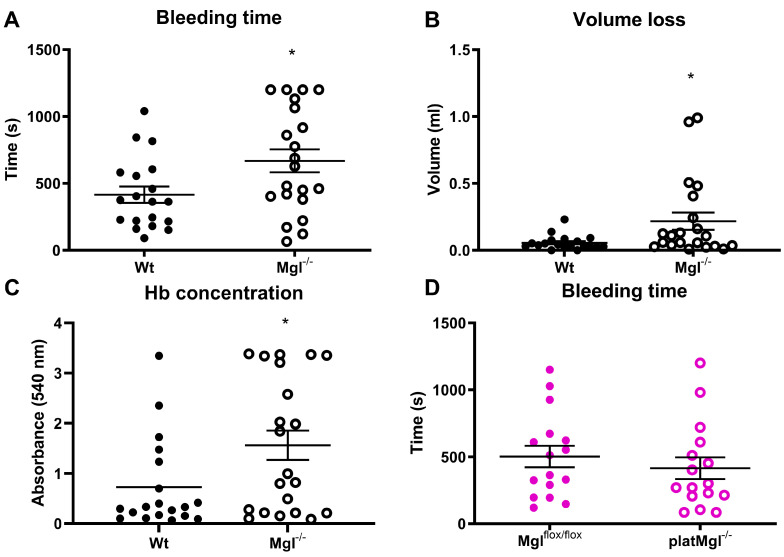

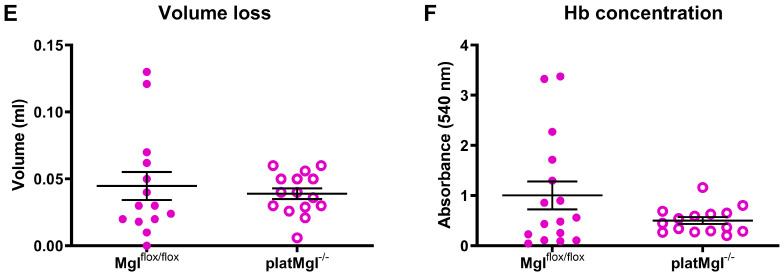
Decreased hemostatic function in Mgl^−/−^ mice. (**A**,**D**) Bleeding time, (**B**,**E**) blood volume loss, and (**C**,**F**) hemoglobin (Hb) absorbance (540 nm) determined in tail vein-isolated blood from Mgl^−/−^ and platMgl^−/−^ mice. Data are expressed as mean ± SEM (n = 20, 21 for Mgl^−/−^ and Wt mice and 16 for platMgl^−/−^ and Mgl^flox/flox^ mice, respectively). *, *p* < 0.05. Significance was calculated by unpaired Student’s *t*-test.

The tail vein bleeding assay determines the in vivo hemostatic capacity of mice after vessel injury. Consistent with reduced platelet aggregation, the assays showed increased bleeding time and blood volume loss in Mgl^−/−^ mice (Figure 4A,B), which was also consistent with increased hemoglobin concentrations of the collected blood loss samples of Mgl^−/−^ compared to Wt mice (Figure 4C) despite comparable circulating hemoglobin levels between the genotypes (Figure S3). Whereas hemostatic function was reduced in Mgl^−/−^ platelets, bleeding time as well as volume loss and hemoglobin levels upon tail bleeding time assays were comparable between platMgl^−/−^ and control animals (Figure 4D–F).

### 2.5. Loss of MGL Affects Thrombus Formation In Vivo

**Figure 5:**
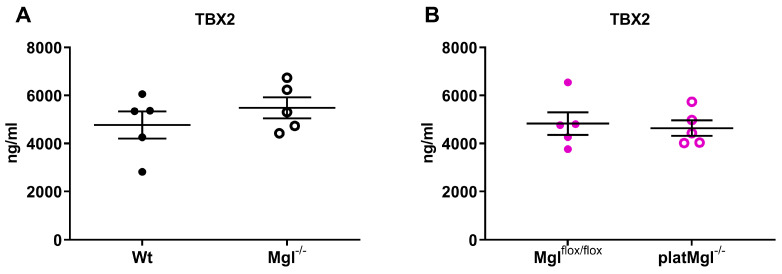

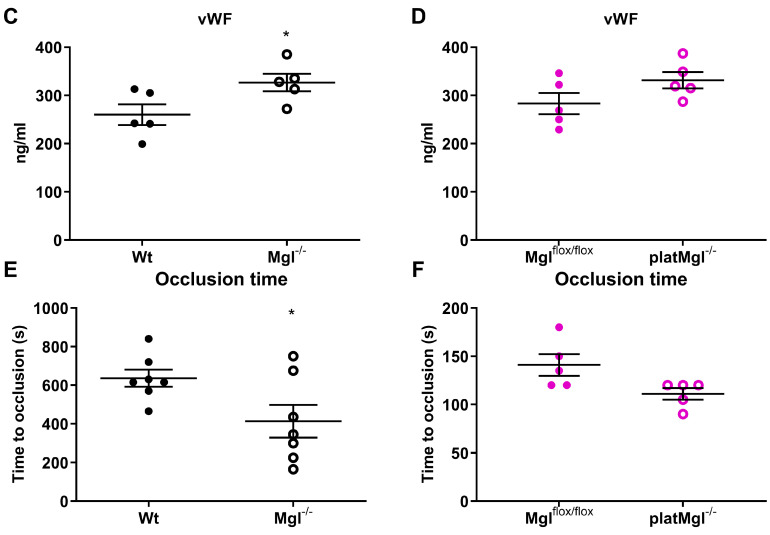
Reduced in vivo occlusion time in Mgl^−/−^ mice. Plasma TXB2 and vWF concentrations were measured by ELISA (mean ± SEM) in plasma from (**A**,**C**) Mgl^−/−^ and (**B**,**D**) platMgl^−/−^ mice (n = 6). Thrombus formation was induced by (**E**) applying a drop of 10% FeCl_3_ in Mgl^−/−^ mice and (**F**) after mechanical injury by a single firm compression with a forceps for 10 s in platMgl^−/−^ mice. Occlusion time was recorded using an ultrasonic flowprobe. Data are expressed as mean ± SEM (n = 5–7). *, *p* < 0.05.

Thromboxane B2 (TXB2), the stable metabolite of thromboxane A2, is produced by activated platelets during platelet plug formation and has pro-thrombotic properties [27]. Accordingly, we investigated whether altered thrombus formation resulted from decreased TXB2 levels, since MGL deficiency may cause reduced availability of AA for lipid mediator production [28]. However, plasma TXB2 concentrations were unchanged in Mgl^−/−^ and platMgl^−/−^ mice (Figure 5A,B), indicating that the majority of AA is released from PC by PLA_2_ activation. Von Willebrand Factor (vWF) levels were slightly increased in Mgl^−/−^, but not in platMgl^−/−^ platelets (Figure 5C,D). Finally, we assessed the thrombosis propensity (representing occlusion time of the carotid artery in vivo) using the FeCl_3_-induced thrombus formation model [29,30]. Occlusion time after FeCl_3_ administration was markedly reduced in Mgl^−/−^ mice (Figure 5E), consistent with the in vitro thrombus formation results of brighter, more contracted larger aggregates and fewer small aggregates (Figure 3C–E). No differences in occlusion time were observed in platMgl^−/−^ mice, neither after FeCl_3_-induced nor after mechanical injury of the carotid artery by a single firm compression with a forceps for 10 s (Figure 5F).

## 3. Discussion

Data from genome-wide RNA-sequencing in humans identified MGL in platelets [22], and a recent study confirmed MGL enzymatic activity in platelets from female and male volunteers [31]. Our previous results in mice [23] also demonstrated high platelet MGL mRNA and protein expression. As a consequence of MGL deficiency, various MG species, including the AG species (MG 20:4), were highly increased in platelets from Mgl^−/−^ mice. Although fatty acid amide hydrolase (FAAH) and α/β-hydrolase domain containing (ABHD)-6 and -12 are capable of degrading 2-AG [32,33,34], the substantial accumulation of multiple MG species in Mgl^−/−^ platelets suggests they are unlikely limiting enzymes in AG degradation in platelets.

Platelets circulate in the bloodstream under physiological conditions, whereas endothelial injury leads to the exposure of subendothelial extracellular matrix proteins and triggers platelet activation and aggregate formation via a multistep process [35]. This includes the release of second-wave mediators that act as soluble agonists such as ADP, TXA2, or the generation of thrombin on activated platelets, which in turn triggers platelet activation via G-protein-coupled receptors [36]. Collectively, these events provide a stimulus for the conformational change of platelet αIIbβ3 integrin (GPIIb/IIIa receptor), which (in its activated form) binds fibrinogen and vWF, thereby triggering platelet aggregation and consequently stable thrombus formation [36,37]. Apart from its reservoir in endothelial Weibel–Palade bodies, vWF is also stored in platelet α-granules, from which it is released following platelet activation [38]. As vWF helps platelets adhere to subendothelial collagen and form a stable thrombus, it is conceivable that increased vWF concentrations in the plasma of Mgl^−/−^ mice is a sign of endothelial perturbation and might partially compensate for the reduced platelet aggregation in vivo.

Platelet activation by 2-AG [16] resembles AA-induced aggregation in human blood and platelet-rich plasma, and includes shape change, aggregation, and ATP secretion [14]. This might be a consequence of 2-AG cleavage, AA release, and/or eicosanoid generation since the selective MGL inhibitor JZL184 diminished the observed effects. Reduced platelet aggregation in response to collagen, probably due to impaired 2-AG degradation, and increased bleeding time in Mgl^−/−^ mice indicate decreased hemostatic function upon MGL deficiency. This finding is consistent with pharmacological MGL inhibition, which is associated with reduced platelet activity [14]. The authors proposed that decreased cleavage of 2-AG reduces AA concentrations, resulting in decreased formation of the pro-thrombotic lipid mediator TXA2 [14].

In line with reduced hemostatic function, platelets from Mgl^−/−^ mice form fewer but larger and brighter (likely with more platelets per thrombus) thrombi on the collagen-coated surface, resulting in a slightly reduced thrombus-covered area in vitro. Accordingly, reduced thrombus formation results in an increased tail bleeding time due to reduced platelet aggregation and thrombus formation. However, this effect only occurs in Mgl^−/−^ but not in platMgl^−/−^ platelets. The in vivo results in the FeCl_3_ carotid thrombosis model, however, were not consistent with the flow experiments as they revealed a faster vessel occlusion in Mgl^−/−^, but not in platMgl^−/−^ mice. This discrepancy between the results might be due to different pathways that can induce thrombosis: the FeCl_3_ model is based on oxidative damage of the vascular cells [39] and a combination of multiple factors in the vessel to activate platelets, whereas the in vitro model detects collagen-related thrombosis. Thus, mice lacking MGL specifically in platelets are protected from pathological bleeding and reduced platelet aggregation, which may be the consequence of lipid uptake by the platelets from circulating lipoproteins [40], leading to unchanged hemostasis in platMgl^−/−^ mice.

Impaired platelet activation may not be due to mitochondrial dysfunction in Mgl^−/−^ platelets because they exhibit increased rather than decreased maximal OCR. Increased mitochondrial respiration may be caused by a variety of factors, including increased platelet activation [26], Ca^2+^ influx, increased energy demand, proinflammatory signaling, proper mitochondrial morphology, and cytoskeletal organization. The latter was shown to be essential for functional mitochondrial respiration via an interplay of various actin-regulating proteins [41]. Reduced mitochondrial respiration resulted in decreased platelet activation [26], leading us to conclude that a lack of platelet function is independent of mitochondrial respiratory capacity in Mgl^−/−^ platelets. However, because of the multiple influences of mitochondrial function, this interesting aspect needs to be investigated in further studies.

The rearrangement of FA in PL, including the acylation of lysophospholipids [42,43] and changes in phospholipid distribution are important processes during platelet activation [44]. Increased Cer levels associate with impaired platelet function [45], which is in line with our findings of higher Cer concentrations and decreased hemostatic function in Mgl^−/−^ platelets. Increased PE concentrations and a trend to increased PC, LPC, and LPE concentrations in Mgl^−/−^ platelets indicate altered membrane composition, which might lead to the observed reduced aggregation and/or activity of Mgl^−/−^ platelets. Analysis of PC and PE species revealed that all species esterified with FA up to 18:2 were increased, whereas the ones esterified with 20:4 and 22:6 FA were decreased. These changes were also observed in LPC and LPE, although less pronounced, indicating a reduced availability of unsaturated FA in platelets from Mgl^−/−^ mice. Platelet activation is one of the main features accompanying the atherosclerotic process. Activated circulating platelets and platelet–leukocyte/monocyte aggregates promote the formation of atherosclerotic lesions in mice [46]. This effect was attributed to platelet P-selectin-mediated delivery of platelet-derived pro-inflammatory factors to monocytes/leukocytes and the vessel wall of ApoE^−/−^ mice [46]. Mgl^−/−^/ApoE^−/−^ mice showed improved atherosclerosis with increased plaque stability and reduced foam cell formation despite larger lesion size [21]. Whether reduced platelet activation in Mgl^−/−^ mice might be involved in the beneficial effect on atherogenesis remains to be elucidated.

The present study shows that, despite comparable platelet morphology, global MGL deficiency is associated with decreased platelet aggregation, with a lower response to collagen activation, and with a phenotype of impaired hemostasis. This was reflected in a reduced number of thrombi in vitro, accompanied by increased tail bleeding time and higher blood volume loss. We conclude that MGL and its degradation products are involved in platelet activation, and aggregation and that loss of systemic MGL impairs collagen-induced thrombogenesis in vitro. Changes in function were absent in platelets from platMgl^−/−^ mice, indicating that lipid degradation products or other molecules in the circulation, rather than platelet-specific effects, may be responsible for the observed alterations in Mgl^−/−^ mice.

## 4. Materials and Methods

### 4.1. Animals

Global Mgl^−/−^ mice were generated as described elsewhere [20]. Mice with a targeted deletion of Mgl in platelets (platMgl^−/−^ mice) were obtained by crossing Mgl^flox/flox^ mice (kindly provided by R. Zimmermann, University of Graz, Graz, Austria) [47] with transgenic mice that express Cre recombinase under the control of the platelet factor 4 promoter (C57BL/6-Tg(Pf4-icre)Q3Rsko/J; Pf4 Cre; C57BL/6 background; kindly provided by B. Nieswandt, University of Würzburg, Würzburg, Germany). Wild-type (Wt) or Mgl^flox/flox^ mice were used as controls. Mice were fed a standard chow diet (4% fat and 19% protein; Altromin Spezialfutter GmbH & Co., Lage, Germany) and water ad libitum on a regular light–dark cycle (12 h light, 12 h dark) in a temperature-controlled environment (22 °C ± 1 °C). All protocols were approved by the Austrian Federal Ministry of Science, Research and Economy, Division of Genetic Engineering and Animal Experiments, Vienna, Austria (BMWF-66.010/0153-WF/V/3b/2015, BMWFW-66.010/0197-WF/V/3b/2017, BMBWF-66.010/0165-V/3b/2019).

### 4.2. Platelet Isolation and Purification

Platelets were isolated as recently described in [23]. To remove residual red blood cells and leukocytes for pure platelet preparations, cells were incubated with anti-Ter-119 and anti-CD45 beads (Miltenyi Biotec, Bergisch Gladbach, Germany), respectively.

### 4.3. Transmission and Scanning Electron Microscopy

For transmission electron microscopy, platelets were fixed in 2% paraformaldehyde/2.5% glutaraldehyde for 1 h, washed, post-fixed in cacodylate buffer/OsO_4_ for 2 h and subsequently washed in cacodylate buffer. After dehydration, samples were infiltrated (propylene oxide and TAAB embedding resin, pure TAAB embedding resin) for 3 h, placed in TAAB embedding resin (2× for 90 min), transferred into embedding molds, and polymerized (72 h, 60 °C). Sections were stained with lead citrate and platinum blue (International Bio-Analytical Industries, Inc., Boca Raton, FL, USA) and investigated with a 120 kV Tecnai G 2 FEI microscope (FEI, Eindhoven, The Netherlands) equipped with a Gatan ultrascan 1000 CCD camera.

For scanning electron microscopy, the platelets were mounted on coverslips to form a thin layer. We then placed platelets on this layer and allowed 1 min for the cells to interact with the surface. The platelets were fixed with 2.5% glutaraldehyde in 100 mM phosphate-buffered saline, pH 7.4, and dehydrated stepwise in a graded ethanol series. Samples were post-fixed with 2% OsO_4_ for 1 h at RT and then dehydrated in graded ethanol series (30–96% and 100% (*v*/*v*) EtOH). Critical point drying (Baltec CPD) and sputter coating (Baltec Sputter Coater 500) were performed. Coverslips were placed on stubs covered with a conductive double-coated carbon tape. Images were acquired using a Sigma 500VP FE-SEM with an SEM Detector (Carl Zeiss, Oberkochen, Germany) at an acceleration voltage of 5 kV.

### 4.4. Targeted Lipidomic Analysis

Cell pellets (in 140 µL dH_2_O) were transferred to 2 mL Safe-Lock PP tubes and lipids were extracted as described [48]. Chromatographic separation was modified according to Knittelfelder et al. [49] as recently described in [23].

MG in purified platelets were analyzed after extraction using a modified protocol as described previously [50]. As internal standard, 266 pmol MG 17:0 (Larodan, Solna, Sweden) was used. Platelets were homogenized at 4 °C for 3 min at 30 Hz (MM 400, Retsch, Haan, Germany). After 30 min of shaking at 4 °C, 200 µL of water was added and shaken for another 20 min. Thereafter, 100 µL of the upper organic phase was dried under constant nitrogen flow and dissolved in 50 µL 2-propanol/methanol/H_2_O (7/2.5/1, *v*/*v*/*v*). UPLC-MS analysis of MG was performed as described previously [50] by changing only the HPLC starting condition of solvent B from 50% to 40%.

### 4.5. Flow Cytometric Analyses of P-Selectin and αIIbβ3 Activation

P-selectin expression and αIIbβ3 activation were determined as previously described [23].

### 4.6. Platelet Aggregation, Tail Bleeding, and Hemoglobin Assays

Platelet aggregation was determined as previously described [23]. Bleeding assays were performed as described elsewhere [51]. Hemoglobin concentrations in tail vein-isolated blood were measured spectrophotometrically as previously described [23]. Hemoglobin concentrations in whole blood were determined using the automated Cell Counter Analyzer MS9-5V (Melet Schloesing Laboratories GmbH, Maria Enzersdorf, Austria).

### 4.7. Mitochondrial Respiration Measurement

Ten million platelets per well were seeded in an Agilent Seahorse XF96 Cell-Tak-coated microplate and oxygen consumption rate (OCR) was measured on an XF96 extracellular flux analyzer (Seahorse Bioscience, North Billerica, MA, USA) as recently described [23].

### 4.8. Thrombus Formation In Vitro

Vena8Fluoro+ biochips (Cellix, Dublin, Ireland) were coated with CHRONO-PAR^®^ collagen I (200 µg/mL; Chrono-Log Corp., Haverton, PA, USA) and perfused with whole blood (shear rate of 30 dynes/cm^2^) as previously described [23]. Thrombus formation was recorded every 30 s for 5 min using a Zeiss Axiovert 40 CFL microscope equipped with a Hamamatsu ORCA-03G digital camera (Hamamatsu, Bridgewater, NJ, USA) and analyzed by Cellix VenaFlux software. The area covered by the thrombus was calculated after image analysis using a costumed ImageJ macro. The time-lapse videos were subtracted from the background and thresholded in parallel using auto-Li and auto-local-Bernsen thresholds. Afterwards, both thresholds were combined to achieve a more stringent and accurate segmentation of the adhesive cells compared to the individual thresholding approaches. The segmented particles were used as a mask to measure thrombus count and thrombus-covered area.

### 4.9. Thrombus Formation In Vivo

The right carotid artery was exposed through a midline incision in the neck and an ultrasonic flowprobe (0.5PSB699; Transonic Systems) was placed around the vessel. For analysis after FeCl_3_-induced injury, thrombosis was induced by topical application of a 0.5-mm^2^ filter paper saturated with 10% FeCl_3_ for 60 s. For mechanical injury, thrombus formation was induced by a single firm compression with a forceps for 10 s. Blood flow was monitored for 30 min or until full occlusion (>5 min) of the vessel occurred [30].

### 4.10. Statistical Analysis

Statistical analyses were performed using GraphPad Prism 5.0 software. Significance was determined by unpaired Student’s *t*-test, ANOVA followed by Bonferroni correction, or Mann–Whitney U test if data were not normally distributed according to the Shapiro–Wilk test. Data are presented as mean values ± SEM or median ± IQR. Floating bars also show minimum and maximum values. The following levels of statistical significance were used: * *p* < 0.05, ** *p* ≤ 0.01, *** *p* ≤ 0.001.

## Data Availability

The data presented in this study are available on reasonable request from the corresponding author. Reagents and detailed methods of all procedures are provided in Section 4 of this manuscript or cited accordingly.

## Supplementary Materials

The following supporting information can be downloaded at: https://www.mdpi.com/article/10.3390/ijms24043116/s1.
