# Supplementary material for: Monoglyceride Lipase Deficiency Is Associated with Altered Thrombogenesis in Mice
Source: Int J Mol Sci. 2023 Feb 4;24(4):3116. doi: 10.3390/ijms24043116 (PMC9958834; doi:10.3390/ijms24043116)
Supplement: Supplementary file 1 [file ijms-24-03116-s001.zip › ijms-2163332-supplementary.pdf]

Supplemental information

Monoglyceride lipase deficiency is associated with altered thrombogenesis in mice

Madeleine Goeritzer, Katharina B. Kuentzel, Sarah Beck, Melanie Korbelius, Silvia Rainer, Ivan Bradić, Dagmar Kolb, Marion Mussbacher, Waltraud C. Schrottmaier, Alice Assinger, Axel Schlagenhauf, René Rost, Benjamin Gottschalk, Thomas O. Eichmann, Thomas Züllig, Wolfgang F. Graier, Nemanja Vujić, and Dagmar Kratky

Figure S1

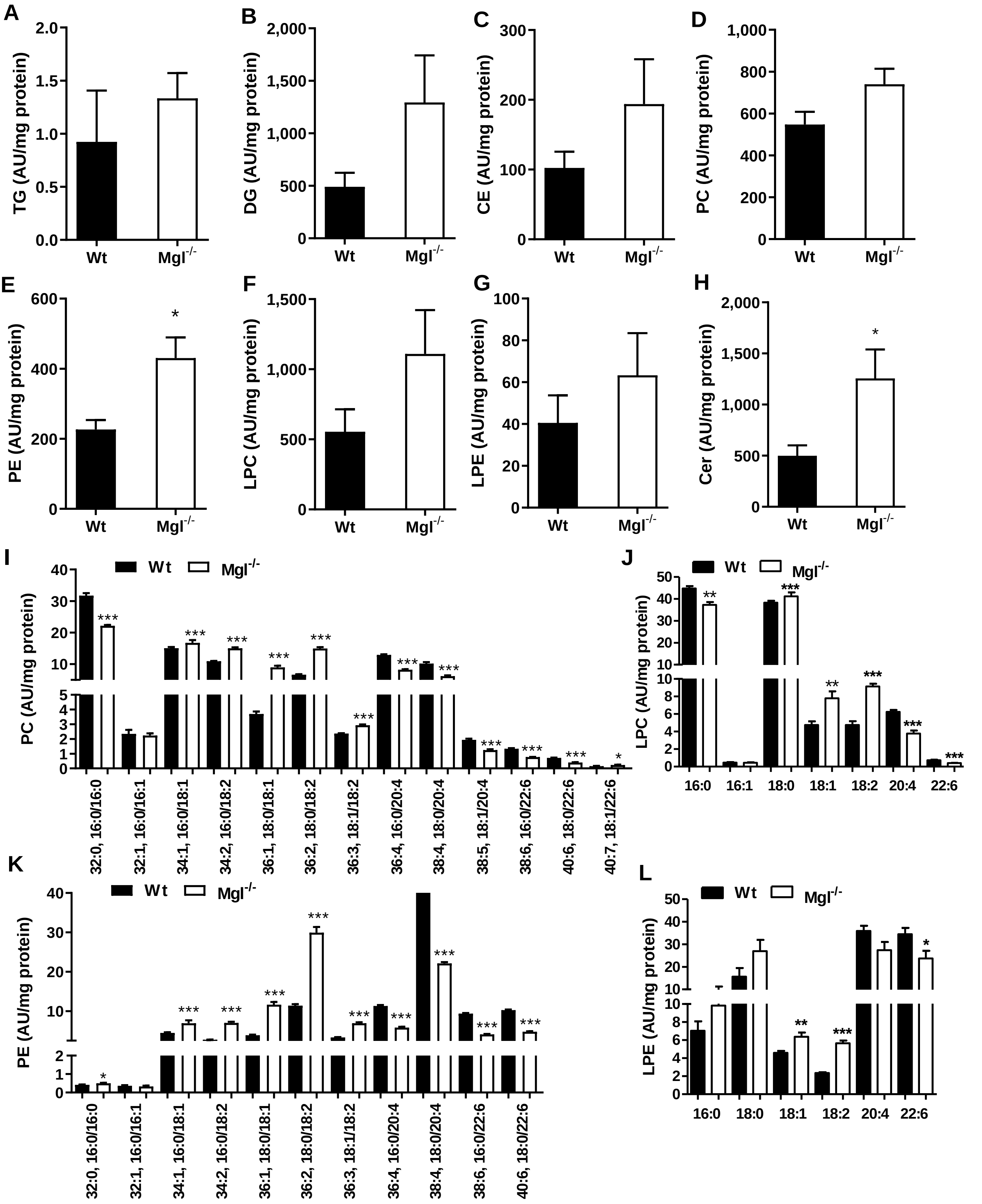
A
TG (AU/mg protein)
Wt
Mgl-/-
B
DG (AU/mg protein)
C
CE (AU/mg protein)
D
PC (AU/mg protein)
E
PE (AU/mg protein)
F
LPC (AU/mg protein)
G
LPE (AU/mg protein)
H
Cer (AU/mg protein)
I
PC (AU/mg protein)
32:0, 16:0/16:0
32:1, 16:0/16:1
34:1, 16:0/18:1
34:2, 16:0/18:2
36:1, 18:0/18:1
36:2, 18:0/18:2
36:3, 18:1/18:2
36:4, 16:0/20:4
38:4, 18:0/20:4
38:5, 18:1/20:4
38:6, 16:0/22:6
40:6, 18:0/22:6
40:7, 18:1/22:6
J
LPC (AU/mg protein)
16:0
16:1
18:0
18:1
18:2
20:4
22:6
K
PE (AU/mg protein)
L
LPE (AU/mg protein)

Figure S1: Increased phospholipid concentrations in platelets from Mgl$^{-/-}$ mice.

Lipids extracted from platelets (pooled from 5 mice) were analyzed by UPLC-MS to quantify (**A**) triacylglycerol (TG), (**B**) diacylglycerol (DG), (**C**) cholesteryl ester (CE), (**D**) phosphatidylcholine (PC), (E) phosphatidylethanolamine (PE), (**F**) lysophosphatidylcholine (LPC), (**G**) lysophosphatidylethanolamine (LPE), and (**H**) ceramide (Cer) concentrations. The levels of individual species in (**I**) PC, (**J**) LPC, (**K**) PE, and (**L**) LPE were calculated. Data are expressed as mean +SEM (n=5). *, $p < 0.05$, **$p < 0.01$, ***$p < 0.001$.

Figure S2

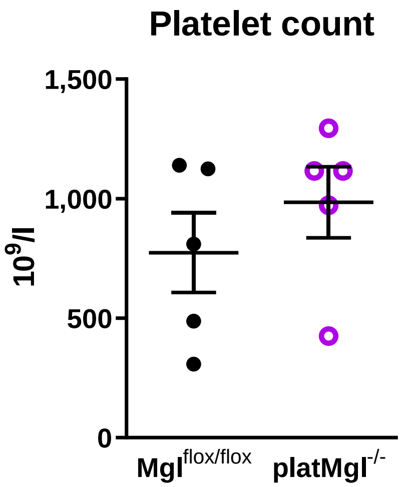

Figure S2: Comparable platelet count in platMgl$^{-/-}$ mce.
Platelet counts in blood from Mgl$^{flox/flox}$ and platMgl$^{-/-}$ mice were measured using an automated cell counter. Data represent single values and mean ±SEM (n=5).

Figure S3

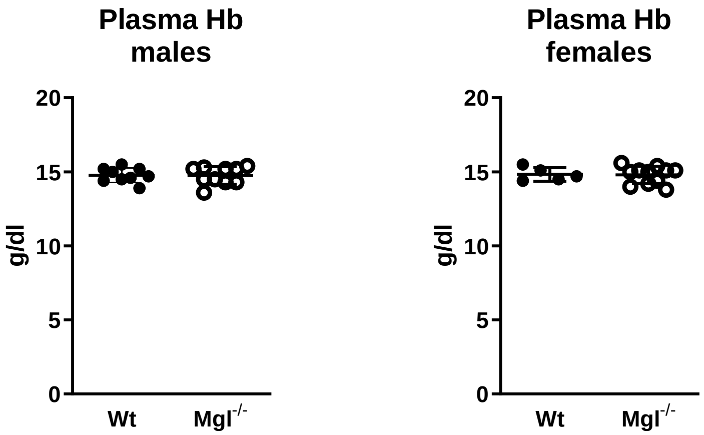

Figure S3: Comparable plasma hemoglobin concentrations in $Mgl^{-/-}$ mice.
Hemoglobin (Hb) concentrations in the plasma from male and female $Mgl^{-/-}$ and Wt mice were measured using an automated cell counter. Data are expressed as mean +SD (n=5-11).
